# Microbial Pathogens Trigger Host DNA Double-Strand Breaks Whose Abundance Is Reduced by Plant Defense Responses

**DOI:** 10.1371/journal.ppat.1004030

**Published:** 2014-04-03

**Authors:** Junqi Song, Andrew F. Bent

**Affiliations:** Department of Plant Pathology, University of Wisconsin - Madison, Madison, Wisconsin, United States of America; Michigan State University, United States of America

## Abstract

Immune responses and DNA damage repair are two fundamental processes that have been characterized extensively, but the links between them remain largely unknown. We report that multiple bacterial, fungal and oomycete plant pathogen species induce double-strand breaks (DSBs) in host plant DNA. DNA damage detected by histone γ-H2AX abundance or DNA comet assays arose hours before the disease-associated necrosis caused by virulent *Pseudomonas syringae* pv. *tomato*. Necrosis-inducing paraquat did not cause detectable DSBs at similar stages after application. Non-pathogenic *E. coli* and *Pseudomonas fluorescens* bacteria also did not induce DSBs. Elevation of reactive oxygen species (ROS) is common during plant immune responses, ROS are known DNA damaging agents, and the infection-induced host ROS burst has been implicated as a cause of host DNA damage in animal studies. However, we found that DSB formation in Arabidopsis in response to *P. syringae* infection still occurs in the absence of the infection-associated oxidative burst mediated by *AtrbohD* and *AtrbohF*. Plant MAMP receptor stimulation or application of defense-activating salicylic acid or jasmonic acid failed to induce a detectable level of DSBs in the absence of introduced pathogens, further suggesting that pathogen activities beyond host defense activation cause infection-induced DNA damage. The abundance of infection-induced DSBs was reduced by salicylic acid and *NPR1*-mediated defenses, and by certain *R* gene-mediated defenses. Infection-induced formation of γ-H2AX still occurred in Arabidopsis *atr/atm* double mutants, suggesting the presence of an alternative mediator of pathogen-induced H2AX phosphorylation. In summary, pathogenic microorganisms can induce plant DNA damage. Plant defense mechanisms help to suppress rather than promote this damage, thereby contributing to the maintenance of genome integrity in somatic tissues.

## Author Summary

Multicellular organisms are continuously exposed to microbes and have developed sophisticated defense mechanisms to counter attack by microbial pathogens. Organisms also encounter many types of DNA damage and have evolved multiple mechanisms to maintain their genomic integrity. Even though these two fundamental responses have been characterized extensively, the relationship between them remains largely unclear. Our study demonstrates that microbial plant pathogens with diverse life styles, including bacteria, oomycete and fungal pathogens, induce double-strand breaks (DSBs) in the genomes of infected host plant cells. DSB induction is apparently a common feature during plant-pathogen interactions. DSBs are the most deleterious form of DNA damage and can lead to chromosomal aberrations and gene mutations. In response to pathogen infection, plant immune responses are activated and contribute to suppressing pathogen-induced DSBs, thereby maintaining better genome integrity and stability. The findings identify important ways that the plant immune and DNA damage repair responses are interconnected. Awareness of the above phenomena may foster future development of disease management approaches that improve crop productivity under biotic stress.

## Introduction

Organisms continuously encounter many types of DNA damage and have evolved elegant mechanisms to maintain their genomic integrity [1], [2]. DNA damage can be induced by a variety of exogenous stresses such as ultraviolet light or genotoxic chemicals, and by endogenous insults such as reactive oxygen species and DNA replication errors [1]–[3]. DNA double-strand breaks (DSBs) can trigger cell cycle arrest and programmed cell death, and are among the most serious types of DNA damage. Surveillance for DSBs and signaling in response to DSBs are therefore critical for cells to orchestrate DNA repair pathways not only in the germ line but also in somatic tissues, to sustain genome stability and survival of the organism [1], [2].

Pathogen management of their own (microbial) DNA integrity has a long history of study [4], as does the study of interactions between viruses and host DNA damage repair processes [5]. There have been far fewer reports or studies of damage to host DNA caused by microbial pathogens. However, it has recently been established that microbial pathogens of animals can induce host DNA damage [6]–[12].

Multicellular organisms are continuously exposed to microbes and have developed effective immune systems to resist attacks by pathogens [13], [14]. Organisms are challenged to balance the health-promoting impacts of antimicrobial responses and the potential toxic effects on surrounding tissue caused by excessive or chronic inflammation. In animal pathogenesis studies, carcinogenic effects of innate immune responses mediated by Toll-like receptors have been reported [15]. An oxidative burst is a common element of plant and animal antimicrobial responses [14], [16], [17], but reactive oxygen species (ROS) also have well-known DNA damaging activities [3], [18]. There is evidence that a significant component of the host genotoxicity of certain microbial infections in animals is attributable to host-generated ROS [8]–[11]. In plants, the relative contribution of the defense-associated ROS burst to pathogen restriction as opposed to genotoxicity (DNA damage) remains to be explored.

Plant DNA damage repair pathways have received extensive study [19], [20]. Although the tie-ins of plant DNA damage to other aspects of organismal physiology are often plant-specific, many elements of the plant DNA damage repair pathways resemble those of animals due to conservation of core DNA damage repair mechanisms [19], [20]. A hallmark DNA damage response conserved across multicellular organisms is the rapid phosphorylation of histone variant H2AX in the chromatin that flanks break sites, forming γ-H2AX [21], [22]. The phosphatidylinositol 3-kinase-like kinases ATM (ataxia telangiectasia mutated) and ATR (ataxia telangiectasia mutated and Rad3-related) are central mediators of these and other cellular responses to DSBs [23]–[25]. γ-H2AX is one of the most sensitive indicators of DNA DSBs [26].

Associations between DNA damage and plant immune responses have been identified. Exposure of plants to the salicylic acid analogs BTH or INA, or inoculation with the oomycete pathogen *H. arabidopsidis*, was shown to increase the frequency of somatic homologous recombination [27]. Infection of tobacco and Arabidopsis leaves with either tobacco masaic virus or oilseed rape mosaic virus (ORMV) results in both local and systemic increases in homologous recombination frequency, and ORMV inoculations elicited DNA damage [28], [29]. DNA damaging agents induce pathogenesis-related gene expression [30], [31], and the DNA damage repair proteins RAD51D, BRCA2 and SSN2 are now known to be involved in regulation of gene expression during plant immune responses [32]–[34]. Poly(ADP-ribosyl)ation, a process frequently associated with DNA damage repair, has been shown to impact plant responses to microbial pathogens [35], [36]. Lastly, during the programmed cell death of the hypersensitive response to pathogens expressing effectors (*Avr* gene products) recognized by a corresponding plant *R* gene product, increased signal in TUNEL (terminal deoxynucleotidyl transferase-mediated dUTP nick end labeling) assays can be observed, as is also common in animal cell apoptosis [37], [38]. In spite of the above, DNA damage during plant interactions with virulent pathogens is largely undescribed, and whether DNA damage arises during responses activated by core plant defense mediators such as salicylic acid, jasmonic acid or activated microbe-associated molecular pattern (MAMP) receptors also is not known.

Here, we demonstrate that diverse microbial pathogens induce DNA double-strand breaks in host plant genomes. Surprisingly (in light of the mutagenic nature of ROS in many settings), infection-associated *AtrbohD*- and *AtrbohF*-dependent ROS production is not required for pathogen-induced elevation of γ-H2AX. Instead, we find that plant antimicrobial defense mechanisms contribute to suppressed formation and/or rapid repair of γ-H2AX-associated lesions. DNA DSB damage is apparently a common aspect of plant pathogenesis by virulent microbial pathogens, and protection against DNA damage is an important feature of effective plant disease resistance.

## Results

### Induction of DNA double-strand breaks by bacterial pathogens

**Figure 1:**
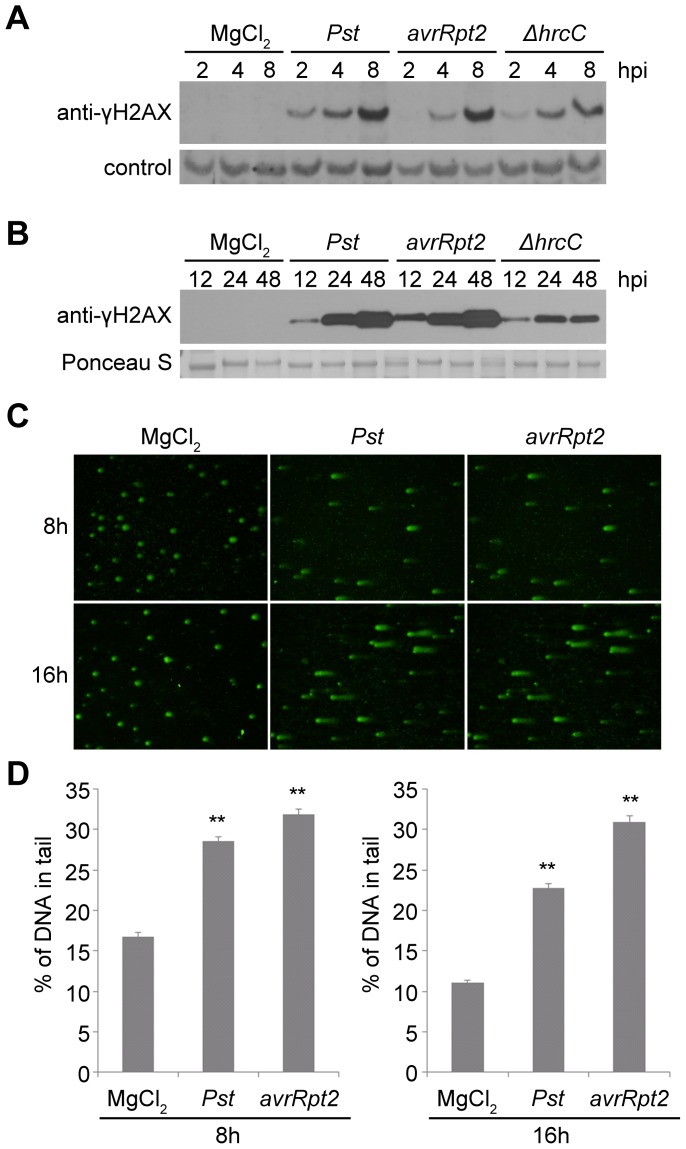
Host DNA damage by *Pseudomonas syringae* pv. *tomato* (*Pst*). (A–B) Accumulation of γ-H2AX during infection. Wild-type Arabidopsis Col-0 plants were vacuum-inoculated with (left to right) 10 mM MgCl_2_, *Pst* DC3000, *Pst* DC3000(*avrRpt2*) or *Pst* DC3000(Δ*hrcC*) at 1×10^7^ cfu/ml. The level of γ-H2AX was monitored at (A) 2, 4, 8 h, or (B) 12, 24, 48 h after inoculation, by immunoblot using anti-γ-H2AX antibody. Controls for equivalent loading included a non-specific band detected by the antibody (control) or Ponceau S staining of the same blot. Similar results were obtained in at least three separate experiments. (C) Representative *Pst*-induced DNA damage detected by comet assay. Wild-type Col-0 plants were inoculated with 10 mM MgCl_2_, or with *Pst* DC3000 or *Pst* DC3000(*avrRpt2*) at 1×10^7^ cfu/ml. Tissues were collected 8 or 16 h after inoculation and nuclei were subjected to comet assays. (D) Comet assay data presented as mean ± SE from at least 200 randomly selected nuclei for each treatment; data for 8 and 16 h are from separate experiments. **: significantly different from MgCl_2_-treated control (ANOVA *P*<0.01).

To investigate interactions between pathogen infection and genome stress, we used γ-H2AX [21], [26] to monitor the extent of DNA damage in response to bacterial pathogens in Arabidopsis. Wild-type Arabidopsis Col-0 plants were challenged with virulent *Pseudomonas syringae* pv. *tomato* (*Pst*) strain DC3000 and levels of γ-H2AX at various time points after infection were determined using an anti-γ-H2AX antibody. Accumulation of γ-H2AX was readily detected as early as 2 h after infiltration, with a progressive increase at the indicated time points after infiltration (Figures 1A and 1B). No γ-H2AX accumulation was observed after mock treatment with 10 mM MgCl_2_, suggesting that the elevated levels of DNA damage were triggered by the pathogen rather than by any physical perturbations associated with plant inoculation.

We also measured the phosphorylation of H2AX in response to *Pst* DC3000(*avrRpt2*), a strain that is isogenic with *Pst* DC3000 except for its expression of the effector AvrRpt2. AvrRpt2 induces a strong host resistance response (an *R* gene-mediated incompatible interaction) in plants that express the resistance gene *RPS2* [39], [40]. Across four independent experiments, the induction of γ-H2AX levels between 2 and 48 h after inoculation was relatively similar between *Pst* DC3000(*avrRpt2*) and *Pst* DC3000. At the early 2, 4 and 8 h time points, minor differences between the two strains in the γ-H2AX levels induced at the same time after inoculation were not reproducible across experiments. To determine whether the high γ-H2AX accumulation after infection is related to the suite of virulence-promoting bacterial effectors delivered via type III secretion, Arabidopsis Col-0 plants were infected with *Pst* DC3000(Δ*hrcC*) that carries a deletion in the *hrcC* gene that encodes a key component of the type III secretion system [41]. Decreased γ-H2AX accumulation was observed with *Pst* DC3000(Δ*hrcC*) 4 to 8 h after infiltration compared with virulent *Pst* DC3000 treatment, and the difference became statistically significant at 24 and 48 h after infiltration (Figures 1A, 1B and S1). As points of reference, our group and other laboratories have previously found that the onset of *Pst* DC3000-induced plant cell death is not observed until ∼16–24 h after infection, and *Pst* DC3000(*avrRpt2*)-induced hypersensitive response cell death also occurs relatively late, with an onset ∼14–18 h after inoculation [e.g.], [ 42], [43]–[45]. Electrolyte leakage (an early sign of the resistance response) is first detected 5–6 h after inoculation of resistant Arabidopsis with *Pst* DC3000(*avrRpt2*) [46], and with *Pst* DC3000, cytosolic Ca*^2+^* increases are not observed for at least 150 min after inoculation [47], well after the 2 h time point at which γ-H2AX accumulation is first detected (Figure 1A).

Independent evidence that *P. syringae* pv. *tomato* infection increases DSBs in Arabidopsis was obtained in comet assays (Figure 1C and 1D). Comet assays measure DNA damage by directly monitoring the increased capacity of fractured DNA to electrophoretically migrate out of isolated nuclei [48], [49]. The roughly comparable levels of DNA damage caused by *Pst* DC3000(*avrRpt2*) and *Pst* DC3000 may be a result of infiltration directly into the leaf interior of the relatively high (1×10^7^ cfu/ml) and equivalent populations of the two pathogen strains. However, these initial results also suggested that stress may be imposed on plant DNA by plant defense responses.

**Figure 2:**
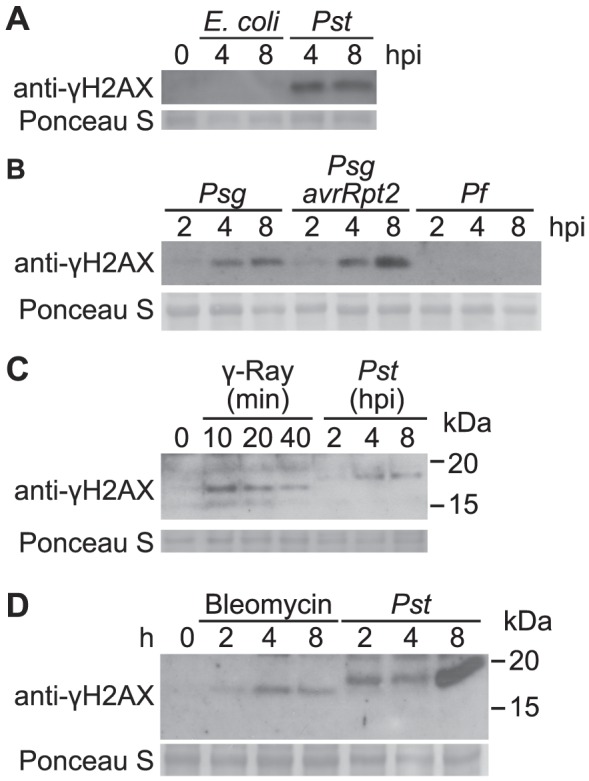
Accumulation of γ-H2AX induced by non-pathogenic pathogens and abiotic stresses. (A) Arabidopsis Col plants were vacuum-infiltrated with an *E. coli* DH5α strain or *Pst* DC3000 at a concentration of 1×10^7^ cfu/ml. The level of γ-H2AX was assessed at 0, 4 and 8 h postinoculation by immunoblot using anti-γ-H2AX antibody. (B) Arabidopsis Col plants were vacuum-infiltrated with *Psg*, *Psg* (*avrRpt2*) or *P. fluorescens* WCS417r at a concentration of 1×10^7^ cfu/ml. The level of γ-H2AX was assessed at 2, 4 and 8 h postinoculation by immunoblot using anti-γ-H2AX antibody. (C) Arabidopsis Col plants were irradiated with 100 Gy of gamma-rays and harvested at 10, 20 and 40 min, or vacuum-infiltrated with *Pst* DC3000 at a concentration of 1×10^7^ cfu/ml and harvested at 2, 4 and 8 h postinoculation. The level of γ-H2AX was assessed by immunoblot using anti-γ-H2AX antibody. (D) Arabidopsis Col plants were treated with 2.5 µg/ml of bleomycin or vacuum-infiltrated with *Pst* DC3000 at a concentration of 1×10^7^ cfu/ml. The level of γ-H2AX was assessed at 0, 2, 4 and 8 h post treatment by immunoblot using anti-γ-H2AX antibody. Equivalent loading of lanes was verified using Ponceau S stain. Similar results were obtained in separate replicate experiments.

A number of non-pathogenic bacteria were then tested for their ability to induce DNA DSBs in the host plant. When wild-type Arabidopsis Col-0 plants were infiltrated with *E. coli* strain DH5α at a dose similar to the above *Pst* inoculations, no γ-H2AX was observed (Figure 2A). Three plant-associated bacterial strains that are not pathogenic on Arabidopsis were also tested: *P. syringae* pv. *glycinea* (*Psg*) is a soybean pathogen that multiplies and produces few visible symptoms when introduced into Arabidopsis leaf mesophyll [50], [51], *Psg* (*avrRpt2*) expresses the effector AvrRpt2 and induces *R* gene-mediated defenses in Arabidopsis Col-0 despite the low virulence of the parent strain [44] and *P. fluorescens* WCS417r is a biological control strain that has been widely used to trigger induced systemic resistance (ISR) in plants [52], [53]. Similar to *E. coli* strain DH5α, *P. fluorescens* WCS417r did not induce detectable levels of γ-H2AX. However, *Psg* and *Psg* (*avrRpt2*) caused elevated phosphorylation of H2AX (Figure 2B).

To compare the extent of *Pst*-induced DSB damage to other known stress conditions, Col-0 plants were irradiated with gamma-rays. Phosphorylation of H2AX was readily detected after exposure to 100 Gy of ionizing gamma irradiation (Figure 2C). Interestingly, the γ-H2AX band induced by *Pst* migrated slightly slower in SDS-PAGE than that induced by gamma-rays, suggesting that additional sites may be phosphorylated upon pathogen infection other than the highly conserved serine at the C-terminus of H2AX protein (Figure 2C). Similarly, when plants were treated with bleomycin, a DNA damage agent that generates DNA DSBs, accumulation of γ-H2AX was induced and a small size difference was observed when compared with the γ-H2AX triggered by *Pst* (Figure 2D).

### Oomycete and fungal pathogens of multiple plant species induce DSBs

**Figure 3:**
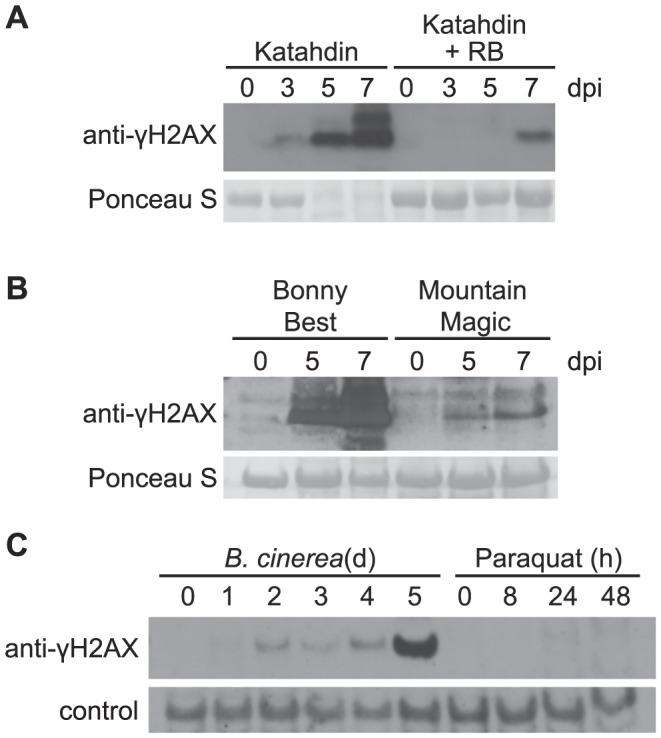
Accumulation of γ-H2AX induced by oomycete and fungal pathogens but not paraquat. The level of γ-H2AX was assessed at indicated times by immunoblot using anti-γ-H2AX antibody. (A) Katahdin and transgenic Katahdin potato plants carrying a single copy of the *RB* gene were spray-inoculated with 5×10^4^ sporangia/ml of a US23 isolate of *P. infestans*. dpi: days post-inoculation. (B) Two tomato varieties, Bonny Best (susceptible) and Mountain Magic (resistant), were spray-inoculated with 5×10^4^ sporangia/ml of a US22 isolate of *P. infestans*. (C) Wild-type Arabidopsis Col-0 plants were spray-treated with either a *Botrytis cinerea* spore suspension (1×10^5^ spores/ml) or 50 µM paraquat, and leaf samples were removed for analysis at the indicated days (d) or hours (h) after treatment. Equivalent loading of lanes was verified using Ponceau S stain or a non-specific band detected by the antibody (control).

To investigate whether DSBs are induced by pathogens other than bacteria, and in other plant species, we examined the level of γ-H2AX in potato and tomato in response to strains of the oomycete pathogen *Phytophthora infestans*. Katahdin, a potato variety susceptible to late blight disease, was challenged with a US23 isolate of *P. infestans*. Figure 3 shows that accumulation of γ-H2AX was induced 3 days after inoculation and significantly increased between 5–7 days after inoculation, a time coincident with visible lesion formation. Extensive γ-H2AX accumulation was similarly detected in tomato variety Bonny Best after inoculation with a US22 isolate of *P. infestans* that is virulent on Bonny Best (Figure 3). At late time points after compatible interactions of potato or tomato with *P. infestans* (i.e., 7 days post-infection) an additional more slowly migrating band was also detected with the anti-γ-H2AX antibody (Figures 3A and 3B). We speculate that H2AX may by that point carry other post-translational modifications [54], [55]. For example, ionizing radiation can induce formation of a ubiquitinated H2AX that migrates at a higher molecular weight and is detected using anti-γ-H2AX antibodies [56].

Incompatible interactions were also tested with these pathogens, to determine if a net increase or decrease in DNA damage is observed relative to compatible interactions in which *R* gene-mediated defenses are not prominent. US23 isolates of *P. infestans* are recognized by the product of the *RB* resistance gene, and *RB* mediates a mild hypersensitive response in potato [57], [58]. Much less accumulation of γ-H2AX was detected when the US23 isolate infected the resistant Katahdin SP951 transgenic potato line (Figure 3A), relative to Katahdin lines that lack the single transgene copy of *RB*. In tomato as well (Figure 3B), significantly less γ-H2AX accumulation was observed when the above-noted US22 *P. infestans* isolate was sprayed onto the tomato variety Mountain Magic that carries the *Ph-2* and *Ph-3* loci that confer resistance to *P. infestans* [59]. This is consistent with the finding that TMV triggered systemic activation of homologous recombination is blocked when resistance gene *N* is absent [28].

The inducibility of DSBs by microbial plant pathogens was further examined in Arabidopsis infected by the necrotrophic fungal pathogen *Botrytis cinerea*. γ-H2AX was induced and its presence sustained between 2 and 4 days after inoculation, and then γ-H2AX increased significantly on the 5^th^ day after inoculation (Figure 3C). Host cell death and leaf collapse also became prevalent on the 5^th^ day after inoculation.

### Paraquat-associated plant cell death does not include strong γ-H2AX induction

Virulent *P. syringae*, *P. infestans* and *B. cinerea* all eventually cause tissue necrosis and plant cell death. To investigate the hypothesis that the elevation of DSBs in plants infected with these virulent pathogens is an event common to any dying plant cells, experiments were conducted with paraquat (methyl viologen). Paraquat is an herbicide that blocks photosynthetic electron transport and causes excess superoxide generation leading to plant cell death [60], but we found no evidence of strong DSB induction by paraquat. Paraquat was applied to Arabidopsis in the same experiment described above in which *Botrytis cinerea* induced γ-H2AX accumulation prior to the appearance of necrotic lesions. With application of 50 µM paraquat, leaves started to wilt 8 h after spraying and developed extensive necrotic lesions by 24 h, but only minimal increases in γ-H2AX abundance were observed (Figure 3C). When 5 µM paraquat was misted onto Arabidopsis leaves in a separate experiment, multiple isolated necrotic lesions formed over the next few days but no elevation of γ-H2AX was observed (in contrast to the *Pst*-inoculated positive control; Figure S2).

### Pathogen-triggered ROS are not a primary cause of pathogen-induced DSBs

**Figure 4:**
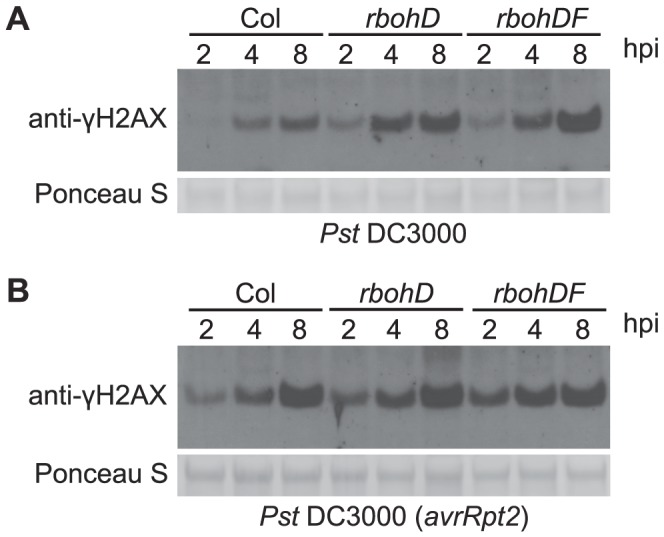
*Pst*-induced γ-H2AX accumulation is independent of *Pst*-triggered ROS production. Four-week old wild-type Arabidopsis Col, *atrbohD* and *atrbohDF* plants were vacuum-infiltrated with (A) *Pst* DC3000 or (B) *Pst* DC3000(*avrRpt2*) at a concentration of 1×10^7^ cfu/ml. The level of γ-H2AX was assessed at 2, 4 and 8 h postinoculation by immunoblot using anti-γ-H2AX antibody. Equivalent loading of lanes was verified using Ponceau S stain.

Elevated ROS are a primary feature of plant defense responses and a possible source of the host DNA damage associated with pathogen infections [3], [17], [18], [61], [62]. Virulent *P. syringae* elicit a rapid but transient accumulation of ROS in plants over approximately the first half hour after infection, while *P. syringae* expressing a recognized avirulence gene induce the first wave as well as a second wave of elevated ROS that is more massive and prolonged [16]. However, ROS production induced by bacterial and oomycete pathogens is nearly eliminated in Arabidopsis *atrbohD* single and *atrbohDF* double mutants with disruptions in the corresponding NADPH oxidase catalytic subunits [17], [63]. We examined the γ-H2AX level in response to pathogen infections in the *atrbohD* and *atrbohDF* mutant plants. There was no obvious reduction of γ-H2AX (Figure 4), in response to either virulent *Pst* DC3000 or avirulent *Pst* DC3000(*avrRpt2*), indicating that pathogen-triggered NADPH-derived ROS production is not the primary cause or a required component of the formation of pathogen-induced DSBs.

### Salicylate-mediated defenses reduce pathogen-induced DSBs

**Figure 5:**
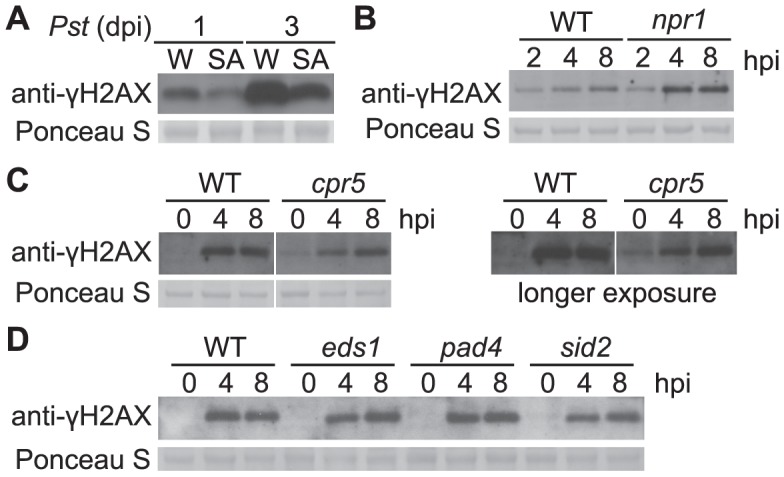
*Pst*-induced γ-H2AX accumulation during salicylic acid signaling and perception. (A) Wild-type Arabidopsis Col-0 plants were pretreated with H_2_O (W) or 1 mM SA for 1 day and then vacuum-inoculated with *Pst* DC3000 at a concentration of 1×10^6^ cfu/ml. The level of γ-H2AX was assessed at 1 and 3 days postinoculation by immunoblot using anti-γ-H2AX antibody. (B) Wild-type Col-0 and Col-0 *npr1* mutant plants were spray-inoculated with *Pst* DC3000 at a concentration of 1×10^7^ cfu/ml. The level of γ-H2AX was assessed at 2, 4 and 8 h postinoculation. (C) Arabidopsis Col-0 and Col-0 *cpr5* mutant plants were vacuum-inoculated with *Pst* DC3000 at a concentration of 1×10^7^ cfu/ml. The level of γ-H2AX was assessed at 0, 4 and 8 h postinoculation by immunoblot using anti-γ-H2AX antibody. Results with both shorter and longer exposure times were shown. (D) Arabidopsis Col-0, *eds1*, *pad4* and *sid2* plants were vacuum-inoculated with *Pst* DC3000 at a concentration of 1×10^7^ cfu/ml. The level of γ-H2AX was assessed at 0, 4 and 8 h postinoculation by immunoblot using anti-γ-H2AX antibody. Equivalent loading of lanes was verified using Ponceau S stain. Similar results were obtained in two additional experiments.

Salicylic acid (SA) is a key signaling molecule that activates defense responses against pathogens in plants, including cellular redox shifts and other physiological responses that could lead to DNA damage [64]–[66]. We investigated if SA induces γ-H2AX accumulation. After wild-type Arabidopsis plants were sprayed with 1 mM SA (a defense-inducing level [67]), no γ-H2AX accumulation was detected at time points up to 48 h after SA treatment (Figure S3). To test if SA-mediated defenses reduce pathogen-induced DNA damage, plants were treated with 1 mM SA for 1 day to induce systemic acquired resistance (SAR), then vacuum-inoculated with virulent *Pst* DC3000. Pretreatment with SA strongly reduced the γ-H2AX accumulation caused by *Pst* DC3000, compared with H_2_O-pretreated controls (Figures 5A and S4A). The finding that SAR reduces *Pst*-induced DNA damage prompted us to investigate the accumulation of γ-H2AX in the SAR-deficient Arabidopsis mutant *npr1*. We detected increased γ-H2AX accumulation induced by *Pst* DC3000 in the *npr1*mutant (Figures 5B and S4B). These data indicate that, rather than causing greater DNA damage, SA-mediated signaling reduces *Pst*-induced damage to host DNA.

Other defense mutants that exhibit altered SA perception and/or signaling were also examined. Arabidopsis *cpr5* plants exhibit constitutive defense responses such as *PR* gene expression, and constitutively elevated levels of SA [68], [69]. A low but constitutive presence of γ-H2AX was detected in the *cpr5* mutant prior to pathogen infection (Figures 5C and S4C). In the Arabidopsis SA signaling mutants *eds1* and *pad4*, and in the SA synthesis mutant *sid2*, no detectable changes of γ-H2AX accumulation after *Pst* infection were observed (Figures 5D and S4D).

We also tested the plant defense signaling molecule jasmonic acid [66]. Similar to SA, jasmonic acid did not induce γ-H2AX accumulation (Figure S3).

### MAMPs do not induce detectable DSBs

We then investigated whether pathogen-free activation of MAMP-induced defense signaling induces γ-H2AX accumulation. The response was monitored from 15 min. to 2 h, which is beyond the half-hour time period when the ROS burst, MAP kinase activation, ethylene synthesis, changes in gene expression and the other primary responses to MAMPs arise [70]. When wild-type Arabidopsis Col-0 seedlings were exposed to 0.1 µM of the bacterial EF-Tu epitope elf18 (a dose sufficient to saturate induction of most elf18-induced plant defense responses [71]), no γ-H2AX accumulation was detected (Figure S5A). The ability of MAMPs to induce γ-H2AX accumulation was also investigated using the flagellin epitope flg22. Again, no γ-H2AX accumulation was observed after a high-dose 1 µM flg22 treatment (Figure S5B), suggesting that typical MAMP-induced plant defense responses do not in general induce sufficient DNA DSBs to cause detectable phosphorylation of H2AX.

### Minimal impact of *sni1*, *ssn2* and *rad51D* mutants on *Pst*-induced DNA damage

**Figure 6:**
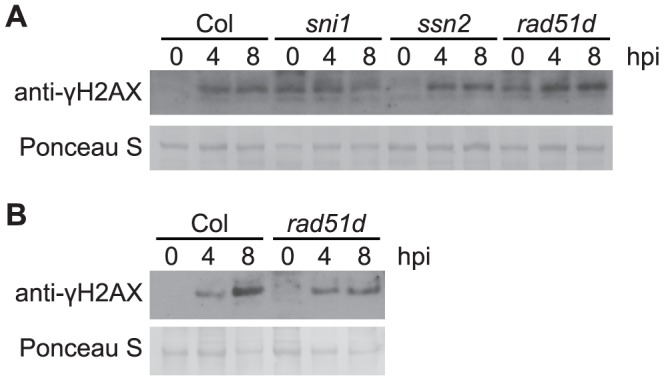
*Pst*-induced γ-H2AX accumulation in mutants involved in homologous-recombination pathway. Wide-type Arabidopsis Col, *sni1, ssn2, rad51d* plants grown under short-day conditions (A), or wild-type Arabidopsis Col and *rad51d* plants grown under long-day conditions (B), were vacuum-inoculated with *Pst* DC3000 at a concentration of 1×10^7^ cfu/ml. The level of γ-H2AX was assessed at 0, 4 and 8 h after inoculation by immunoblot using anti-γ-H2AX antibody. Equivalent loading of lanes was verified using Ponceau S stain. Similar results were obtained in two additional experiments.

The DNA damage (DNA protection) proteins SNI1, SSN2, and RAD51D have been shown to play roles in both homologous recombination and defense gene transcription [32], [34]. To test for a possible contribution of these proteins to prevention or reduction of *Pst*-induced DNA damage, the level of γ-H2AX in response to *Pst* was examined in the respective Arabidopsis Col-0 mutants. SNI1 is a subunit of the Structural Maintenance of Chromosome (SMC) 5/6 complex involved in DNA damage response [72] and functions as a negative regulator of some plant defense responses [32]–[34]. The *sni1* single mutant was recently reported to exhibit a constitutive DNA damage response [72]. Consistent with this, we observed phosphorylation of H2AX in -*sni1* plants in the absence of pathogen infection (Figures 6 and S6A). However, no reproducibly significant changes in the time course of *Pst*-induced γ-H2AX were observed. SSN2 is a SWIM-domain containing protein that acts at early steps of homologous recombination, and the *ssn2* mutation partially suppresses *sni1* [34], [73]. Rad51D complexes with SSN2 and SNI1 during homologous recombination and the *rad51d* mutation also suppresses *sni1* [32]. With *rad51d* and *ssn2* single mutants, we found that the time course of *Pst*-induced γ-H2AX again was comparable to that in wild-type plants (Figures 6 and S6A). When the *rad51d* mutant was grown under short-day conditions, it displayed a spontaneous lesion phenotype and exhibited elevated levels of γ-H2AX without pathogen infection (Figures 6 and S6B).

### ATR and ATM contribute to restriction of virulent and avirulent *Pst* bacteria but are not required for pathogen-induced H2AX phosphorylation

**Figure 7:**
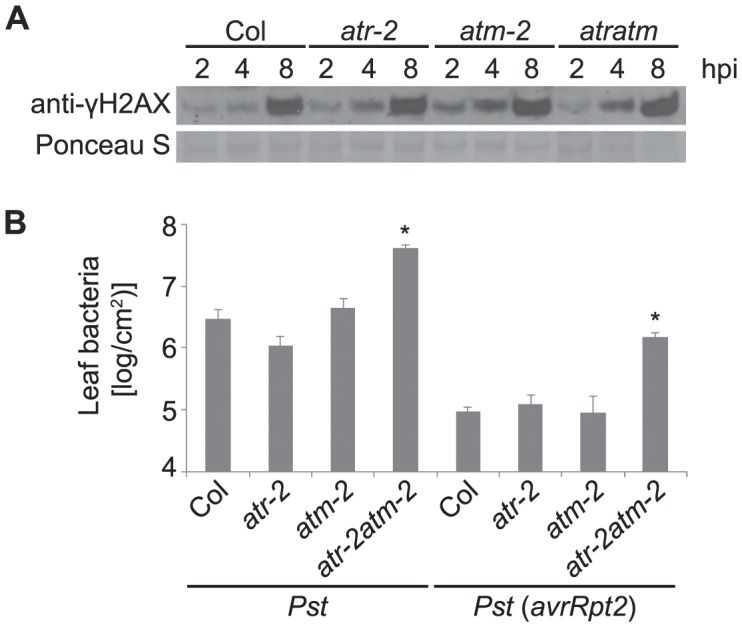
*Pst*-induced γ-H2AX accumulation is independent of ATR and ATM but *atr atm* double mutants are more susceptible to growth of *Pst* bacteria. (A) Wild-type Arabidopsis Col, Col *atr-2* or Col *atm-2* single mutants, or Col *atr-2 atm-2* double mutant plants were vacuum-inoculated with *Pst* DC3000 at a concentration of 1×10^7^ cfu/ml. The level of γ-H2AX was assessed at 2, 4 and 8 h after inoculation by immunoblot using anti-γ-H2AX antibody. Equivalent loading of lanes was verified using Ponceau S stain. Similar results were obtained in three additional experiments. (B) Growth of *Pst* within leaves. Plants were infiltrated with *Pst* DC3000 or *Pst* DC3000(*avrRpt2*) at a concentration of 1×10^5^ cfu/ml. Error bars are SEM for four replicates for each sample within the experiment. * indicates significant difference from Col-0 (ANOVA, Tukey pairwise comparisons, *P*<0.05). Similar results were obtained in two additional experiments.

The accumulation of γ-H2AX after exposure to ionizing radiation is largely dependent on ATM in Arabidopsis, although ATR can contribute to formation of DSBs to a lesser extent [25]. To determine whether Arabidopsis ATR or ATM is required for the phosphorylation of H2AX in response to pathogen infection, we examined γ-H2AX levels in *atr* and *atm* single mutants and in *atr atm* double mutant plants. We used SALK T-DNA insertion lines *atr-2* and *atm-2* in the Col-0 background, that carry an insertion in exon 10 of *ATR* and intron 64 of *ATM* respectively, and have been characterized previously [74], [75]. Similar levels of γ-H2AX were induced by virulent *Pst* DC3000 in the *atr-2* and *atm-2* single mutants and the *atr-2 atm-2* double mutants compared with wild-type Col-0 (Figure 7A). To verify this result we also tested *atr-3* and *atm-1*, which carry in the Ws genetic background a T-DNA insertion in the highly conserved C-terminal kinase domain (*atr-3*) or in the 3′ region of the gene (*atm-1*), and both likely act as null alleles [74]. γ-H2AX induction after *Pst* infection was detected in *atr-3* and *atm-1* single mutants and in *atr-3 atm-1* double mutants at comparable levels to wild-type (Figure S7). Similar results were obtained in two independent experiments with the Col *atr* and *atm* mutants, and in three independent experiments with the Ws *atr* and *atm* mutants. These experiments are consistent with the slightly larger γ-H2AX band observed after *Pst* treatment as opposed to gamma-rays or bleomycin (Figure 2), and suggest that protein kinases other than ATR and ATM are engaged to mediate pathogen-induced γ-H2AX formation.

The response of *atr-2* and *atm-2* mutants to pathogen infection was examined using *Pst* DC3000. The *atr-2 atm-2* double mutants were more susceptible to infection than wild type plants whereas the *atr*-2 and atm-*2* single mutants were similar to wild type (Figure 7B), indicating that ATR and ATM play overlapping roles in basal defense. To test whether ATR and ATM are required for effector triggered immunity, *atr-2* and *atm-2* lines were inoculated with the avirulent pathogen *Pst* DC3000(*avrRpt2*). As shown in Figure 7B, the *atr-2 atm-2* double mutants exhibited enhanced susceptibility. Taken together with previously published findings [74]–[78], these bacterial growth data provide another example that Arabidopsis ATR and ATM can play broad roles in plant development, DNA damage repair, and now, plant immunity.

## Discussion

The present study discovered that host DNA damage is induced, both in the model organism Arabidopsis *thaliana* and in tomato and potato crop plants, in response to plant pathogens with diverse life styles including a hemibiotrophic bacterial species, an oomycete and a necrotrophic fungus. Similar or reduced levels of DNA DSBs were induced during incompatible interactions when compared with compatible interactions. Plant defense mediators such as ROS, jasmonic acid and MAMP receptors did not on their own increase DSBs, and SA-mediated defenses reduced rather than elevated pathogen-induced DNA damage. These findings provide a new type of evidence of links between the plant immune and DNA damage responses. Prevention and repair of DNA damage is needed, to a greater extent than was previously understood, as an element of the plant defense response.

Previously discovered associations between DNA damage and plant immune responses were noted in the introduction [27]–[38]. In addition, Yan et al. very recently reported that salicylic acid activates DNA damage responses as part of the plant immune response [72]. Similar associations have been established in animal systems. For example, DNA damage can regulate human inflammatory responses through activation of the tumor suppressor p53 and elevated expression of Toll-like receptors [79], and the DNA damage response induces expression of innate immune system ligands of the NKG2D receptor [80]. The fact that DNA damage induces immune responses suggests that multicellular organisms associate DNA damage with, among other things, microbial infections. The present work and [29] provide experimental evidence for a key part of this arrangement, by showing that diverse plant pathogens elicit plant DNA damage.

One point of note is the short time after *Pst* infection at which γ-H2AX becomes apparent. The flagellin or EF-Tu MAMPs flg22 or elf18 did not elicit detectable DNA DSBs, but γ-H2AX was reproducibly present within 2 h after *Pst* infection. The pathbreaking findings of [27], [28] indicate that pathogens do not even need to be physically present at a cell for that cell to experience pathogenesis-associated genome stress. They measured homologous recombination rather than directly monitoring DNA damage, but as one example, those researchers reported increased homologous recombination in non-inoculated leaves as early as 8 h after inoculation of tobacco with Tobacco mosaic virus [28]. This is faster than the virus itself moves. The SA analogs BTH or INA induced a 1.5 to 7 fold increase of homologous recombination frequency 14 days after chemical treatment [28], while we did not observe increases in DNA DSBs after treatment with SA or JA. However, we examined the level of γ-H2AX at much earlier times points 12, 24 and 48 h after SA or JA application. Yan et al. reported, from comet assays, that SA can induce DNA damage in wild-type and *npr1* mutant plants [72]. This discrepancy between the two studies may have been caused by the use of different DNA damage detection methods or by differences in plant growth and treatment conditions. The main conclusion of Yan et al. [72], that SA activates DNA damage responses to potentiate plant immunity, is highly consistent with our main finding that pathogenic microorganisms can induce plant DNA damage and that plant defense mechanisms help to suppress rather than promote this damage.

Production of ROS is one of the earliest cellular responses of plants to pathogens and is also a common response to pathogens in animals [14], [16], [17]. The genotoxicity of certain microbial infections in animals has been attributed to host-generated ROS [8]–[11]. We found that Arabidopsis *rbohD* and *rbohDF* mutants that are defective in pathogenesis-induced ROS burst still produced extensive pathogenesis-induced DSBs. In addition, the strong ROS inducer paraquat failed to induce extensive DSBs. Furthermore, despite the contrasting induction of a less strong early ROS burst in response to virulent pathogens vs. the combined early ROS burst and a stronger and more prolonged later ROS burst in response to avirulent pathogens [16], both types of *Pst* DC3000 pathogens induced similar formation of DSBs. Recognition of diverse MAMPs including both flg22 and elf18 also triggers an oxidative burst [70] but failed to induce the generation of DSBs. Collectively, these findings indicate that ROS are not key mediators required for pathogen induction of DSBs in the plant pathosystems that we analyzed. A recent paper has analogously suggested that the host ROS triggered by *H. pylori* infection is not required for DSB formation in animals [7].

Because in our studies the MAMPs flg22 and elf18, the signaling molecules salicylic acid and jasmonic acid, and ROS-generating paraquat each failed to induce detectable level of DSBs, we postulate that direct interaction with one or more pathogen-derived effectors, toxins, or other molecules is required for pathogen induction of DNA damage. Type-III secretion-defective *Pst* DC3000 Δ*hrcC* induced fewer DSBs, suggesting a contribution of one or more Type III-secreted effectors to bacteria-induced plant DNA damage. However, that contribution may be direct, or indirect through effector elicitation of specific host responses, or indirect due to general enhancement of pathogen population sizes. The more significant result of the DC3000 Δ*hrcC* experiments may be that substantial DSB induction was evident even when the Type-III secretion system was disabled.

In the present study, host DNA DSB induction was observed following infection by plant pathogens but not after introduction of non-pathogenic bacteria. Prior work provides some context for this result. For example, the non-pathogenic bacteria *E. coli* DH5α and *P. fluorescens* Pf101 elicit defense transcript accumulation and phytoalexin biosynthesis in bean [81]. Inoculation of Arabidopsis roots with *P. fluorescens* strain WCS417r (the strain used in this study) activates induced systemic resistance, which is independent of SA accumulation and pathogenesis-related (*PR*) gene activation but primes plants to respond faster or stronger to pathogen attack [82], [83]. Induction of those responses, then, is not likely to elicit DNA DSBs, although the failure of those non-pathogenic bacteria to induce DNA DSBs may alternatively be attributable to the weak defense response they trigger. In contrast to *P. fluorescens* WCS417r, the soybean pathogen *Psg* was previously shown to trigger a marked systemic increase of SA level and *PR* gene expression in Arabidopsis, leading to elevated systemic resistance to secondary infection, although to a lesser extent compared to those induced by virulent *Pst* and avirulent *Pst avrRpm1* [50]. So although we do not yet know the mechanism of DNA DSB induction by plant pathogens, and did not observed its induction by ROS, SA, JA, or MAMPs, strength of host defense induction is a feature that correlates with the DNA DSB-inducing behavior of the strains we studied. Recent work with *Psg* on Arabidopsis reminds us that molecular mechanisms of non-host resistance against different plant pathogens can be distinct. The Arabidopsis non-host resistance gene *PSS1* confers a new form of non-host resistance against both a hemibiotrophic oomycete pathogen, *P. sojae* and a necrotrophic fungal pathogen, *F. virguliforme*, but not the bacterial pathogen *Psg* [84].

ATM and ATR are the two primary known signal transducers of DNA breakage, and they initiate a phosphorylation-mediated signal transduction cascade that leads to cell-cycle arrest and repair of DSBs [23]–[25]. In addition to ATM and ATR, a related mammalian enzyme, the DNA-dependent protein kinase (DNA-PK), is capable of phosphorylating H2AX in response to DSBs [85]. The relative roles of ATM and ATR in DSB-dependent γ-H2AX induction have been debated, and apparently vary depending on the biological context. For example, Kuhne *et al.* provided evidence that ATM contributes to ionizing radiation-induced γ-H2AX formation in mouse fibroblasts [86], whereas a separate report suggested that ATM was not required or played minor role in ionizing radiation-dependent γ-H2AX accumulation [87]. In Arabidopsis the accumulation of γ-H2AX in response to ionizing radiation-induced DSBs is dependent on both ATM and ATR, with a predominant role for ATM [25]. We found that pathogen-induced γ-H2AX accumulation was not reduced in two different Arabidopsis *atr atm* double mutant lines. Our finding unsettles the concept that only ATR and ATM carry out this process in plants. Because no obvious homologs of DNA-PK are present in nonvertebrates, our experiments suggest that plants have one or more kinases other than ATM, ATR or DNA-PK that can phosphorylate H2AX, and which do so in response to pathogen infection. This finding of pathogen-induced γ-H2AX accumulation in Arabidopsis *atr atm* double mutants is supported by a recent discovery made using primary cultures of human renal proximal tubule epithelial cells, where knockdown of all three major phosphatidylinositol 3-kinase-like kinases (ATM, ATR, and DNA-PKcs) did not abolish the activation of γ-H2AX during viral infection by BKPyV [88]. Viral infection by BKPyV did cause severe DNA damage in the absence of ATM or ATR [88], and as previously noted, numerous animal and plant studies have shown that ATM and ATR play central roles in DNA damage repair [19], [24], [25]. Hence it is not overly surprising that Arabidopsis *atr atm* double mutant plants exhibit heightened disease susceptibility, even though ATM and ATR are not the sole means through which plant H2AX can be phosphorylated after pathogen infection.

The discovery of pathogen-induced plant DNA damage [present study and 29] opens intriguing avenues for future study. For example, research to pinpoint pathogenesis-induced DSB sites may reveal if preferential sites exist. Investigation of the pathogen factors or pathogen-induced plant factors that lead to infection-associated DNA damage will be a priority, and this may lend insight into disease management mechanisms that can protect the genome from the damage induced by pathogens. The subject has clear implications for improved crop productivity under conditions of biotic stress [19], [20].

## Materials and Methods

### Plant treatments

Arabidopsis plants were grown at 22°C under 9-h light/15-h cycles in Fison's Sunshine Mix #1. The *rad51d* mutant line was also grown at 16-h light/8-h dark cycles. Five-week-old Arabidopsis plants were typically used in various treatments unless otherwise indicated. *Pseudomonas syringae* pv. *tomato* bacterial strains used in this study were *Pst* DC3000, *Pst* DC3000(*avrRpt2*) [39] and *Pst* DC3000(Δ*hrcC*) [41]. Nonpathogenic bacteria strains included in this study were *E. coli* DH5α grown on LB plates, or *P. syringae* pv. *glycinea* (*Psg*), *Psg* (*avrRpt2*) [44] or *P. fluorescens* WCS417r [52] grown on NYGA plates. For DNA damage experiments, above-soil portions of intact plants were briefly inverted into a bacterial solution at 1×10^7^ cfu/ml in 10 mM MgCl_2_, bacteria were introduced into leaf mesophyll by vacuum infiltration [89], plants were returned to their normal growth environment, and samples were collected at the specified time points. For SAR induction, plants were pretreated with 10 mM MgCl_2_ or 1 mM SA for 1 day followed by vacuum-infiltration with virulent *Pst* DC3000 at 1×10^6^ cfu/ml. For ionizing radiation, Arabidopsis Col-0 plants were irradiated at 100 Gy with a ^137^Cs source and collected at indicated times. For bleomycin treatment, Arabidopsis Col-0 plants were incubated with 2.5 µg/ml of bleomycin for indicated times. For *Botrytis cinerea* infection [90], plants were sprayed with 1×10^5^ spores/ml and samples were collected 1 to 5 days post inoculation. Inoculation of *Phytophthora infestans* was carried out by spraying plants with sporangial suspensions according to previously described procedures [57]. Potato and tomato plants were infected with US23 or US22 *P. infestans* isolates, respectively, provided courtesy of Amilcar Sánchez-Pérez and Dennis Halterman. Treatment with paraquat (methyl viologen; Sigma-Aldrich, St. Louis, MO) was performed on Arabidopsis plants by spraying an aqueous suspension to run-off at concentrations of 5 or 50 µM. To obtain *atr atm* double mutants, progeny plants from self-fertilized *atr-3/atr-3,ATM/atm-1* or *atr-2/atr-2,ATM/atm-2* lines [74], [75] were genotyped by PCR for presence of the relevant T-DNA insertion and separately for absence of the wild-type allele using the methods of http://signal.salk.edu/tdnaprimers.2.html. The resulting double mutant lines were observed to be sterile.

### Bacterial growth assay

Five-week-old Arabidopsis seedlings were inoculated with *Pst* DC3000 or *Pst* DC3000(*avrRpt2*) at 1×10^5^ cfu/ml by infiltration of leaf mesophyll using a 1 cc plastic syringe with no needle or vacuum infiltration of immersed rosette leaves. After 3 days, leaf discs were taken from eight inoculated fully expanded rosette leaves and samples from two leaves were combined to form a single replicate and macerated in 10 mM MgCl_2_. The samples were then diluted serially, plated on NYGA plates and colony counts were recorded two days after incubation at 28°C.

### Histone preparations and immunoblotting

Histones were extracted from plant leaf tissue nuclear preparations as previously described [25], [91]. Protein samples were subjected to SDS-PAGE, blotted and immunodetected with rabbit anti-human γ-H2AX antibody at 1∶5000 dilution (Sigma-Aldrich, St. Louis, MO). Band intensity on immunoblots was quantified using Image Studio Lite software version3.1 (LI-COR Biosciences, Lincoln, Nebraska) and statistical tests of significance were performed on the resulting data as described.

### Comet assay

Comet assays [48], [49] were performed using the CometAssay kit from Trevigen (Gaithersburg, MD) with minor modifications. Leaf tissues were cut into pieces with a razor blade in 500 µl 1× PBS buffer supplemented with 20 mM EDTA on ice. Nuclei suspension was filtered into an Eppendorf tube through 50 µm nylon mesh, combined with Comet low-melting-point agarose at a ratio of 1∶ 10 and pipetted onto CometSlides. After incubation in lysis solution for 1 h at 4°C, the slides were placed in 1× Tris-Acetate electrophoresis buffer for 30 min prior to electrophoresis in the same buffer for 10 min at 4°C. Nuclei were stained with SYBR green. Images were captured and quantified with CometScore software (Tritek Co., Sumerduck, VA). At least 200 nuclei were scored per slide.

### Arabidopsis gene numbers

*H2AXa*: AT1G08880; *H2AXb*: AT1G54690; *NPR1*: AT1G64280; *CPR5*: AT5G64930; *EDS1*: AT3G48090; *PAD4*: AT3G52430; *SID2*: AT1G74710; *FLS2*: AT5G46330; *EFR*: AT5G20480; *SNI1*: AT4G18470; *SSN2*: AT4G33925; *RAD51D*: AT1G07745; *ATR*: AT5G40820; *ATM*: AT3G48190.

## Supporting Information

Figure S1 **Host DNA damage by** ***Pseudomonas syringae*** **pv.** ***tomato*** **(** ***Pst*** **) detected by accumulation of γ-H2AX during infection.** Wild-type Arabidopsis Col-0 plants were vacuum-inoculated with (left to right) 10 mM MgCl_2_, *Pst* DC3000, *Pst* DC3000(*avrRpt2*) or *Pst* DC3000(Δ*hrcC*) at 1×10^7^ cfu/ml. The level of γ-H2AX was monitored at 2, 4, 8, 12, 24, 48 h after inoculation, by immunoblot using anti-γ-H2AX antibody. Levels of γ-H2AX detected by immunoblot were quantified and data are shown as mean ± SE from three independent experiments. All time points after treatment with *Pst* DC3000, *Pst* DC3000(*avrRpt2*) or *Pst* DC3000(Δ*hrcC*) showed significant difference from MgCl_2_-treated control (*P*<0.05). * indicates time points at which significant difference observed for treatments with *Pst* DC3000 or *Pst* DC3000(*avrRpt2*) as compared with *Pst* DC3000(Δ*hrcC*) (Student's *t*-test, *P*<0.05).
(EPS)

Figure S2 **Accumulation of γ-H2AX induced by 5 µM paraquat.** Wild-type Arabidopsis Col-0 leaves were spray-treated with 5 µM paraquat. The level of γ-H2AX was assessed at indicated times. As a positive control, other plants were vacuum-inoculated with *Pseudomonas syringae* pv. *tomato* strain DC3000 (*Pst*) at a concentration of 1×10^7^ cfu/ml. Blot stained with Ponceau S as a loading control. See also Figure 3 showing results after 50 µM paraquat treatment.
(EPS)

Figure S3 **No detectable accumulation of γ-H2AX in response to salicylic acid or jasmonic acid.** Five-week old Arabidopsis Col-0 plants were sprayed with 1 mM SA or 50 µM methyl JA and the level of γ-H2AX was examined at indicated times after treatment. + indicates a positive control sample from wild-type plants at 8 h after *Pst* DC3000(*avrRpt2*) treatment. Blots also stained with Ponceau S as a loading control.
(EPS)

Figure S4 ***Pst*** **-induced γ-H2AX accumulation during salicylic acid signaling and perception.** (A) Wild-type Arabidopsis Col-0 plants were pretreated with 1 mM SA or H_2_O for 1 day and then vacuum-infiltrated with *Pst* DC3000 at a concentration of 1×10^6^ cfu/ml. The level of γ-H2AX was assessed at 1 and 3 days postinoculation by immunoblot using anti-γ-H2AX antibody. Wild-type Col-0, *npr1* (B), *cpr5* (C), *eds1*, *pad4* and *sid2* (D) mutant plants were spray-inoculated with *Pst* DC3000 at a concentration of 1×10^7^ cfu/ml. The level of γ-H2AX was assessed at indicated time points by immunoblot using anti-γ-H2AX antibody. Levels of γ-H2AX detected by immunoblot were quantified and data are shown as mean ± SE from three independent experiments. * indicates significant difference from control at same time point (Student's *t*-test, *P*<0.05).
(EPS)

Figure S5 **No detectable accumulation of γ-H2AX in response to MAMPs.** Two-week old Arabidopsis Col-0, *fls2* or *efr* seedlings grown in liquid MS were treated with 0.1 µM elf18 (A) or 1 µM flg22 (B), respectively, and the level of γ-H2AX was examined at indicated times after treatment. + indicates a positive control showing the accumulation of γ-H2AX in wild-type plants at 8 h after *Pst* DC3000(*avrRpt2*) treatment. Blots also stained with Ponceau S as a loading control.
(EPS)

Figure S6 ***Pst*** **-induced γ-H2AX accumulation in mutants involved in homologous-recombination pathway.** Wide-type Arabidopsis Col, *sni1, ssn2, rad51d* plants grown under short-day conditions (A), or wild-type Arabidopsis Col and *rad51d* plants grown under long-day conditions (B), were vacuum-inoculated with *Pst* DC3000 at a concentration of 1×10^7^ cfu/ml. The level of γ-H2AX was assessed at 0, 4 and 8 h after inoculation by immunoblot using anti-γ-H2AX antibody, quantified, and data are shown as mean ± SE from three independent experiments. * indicates significant difference from control treatment at same time point (Student's *t*-test, *P*<0.05).
(EPS)

Figure S7 ***Pst*** **-induced γ-H2AX accumulation is independent of ATR and ATM.** Wild-type Arabidopsis Ws, or *atr-3*, *atm-1* or *atr-3 atm-1* plants were vacuum-inoculated with *Pst* DC3000 at a concentration of 1×10^7^ cfu/ml. The level of γ-H2AX was assessed at 2, 4 and 8 h after inoculation. Blots also stained with Ponceau S as a loading control. Similar experiments with Arabidopsis Col *atr atm* mutants are shown in Figure 7.
(EPS)
