# Correction: Microbial Pathogens Trigger Host DNA Double-Strand Breaks Whose Abundance Is Reduced by Plant Defense Responses

**DOI:** 10.1371/journal.ppat.1004226

**Published:** 2014-06-06

**Authors:** The PLOS Pathogens Staff

Figure 1 in the original article contains two duplicate panels that were inserted during a manuscript revision. Two of the six images in Figure 1C were inadvertently deleted and replaced with duplicates of the adjacent photographic panels. The six correct Figure 1C photographic panels were used during peer review, and are now restored in the corrected version of Figure 1. Please see the correct Figure 1 here.

**Figure 1:**
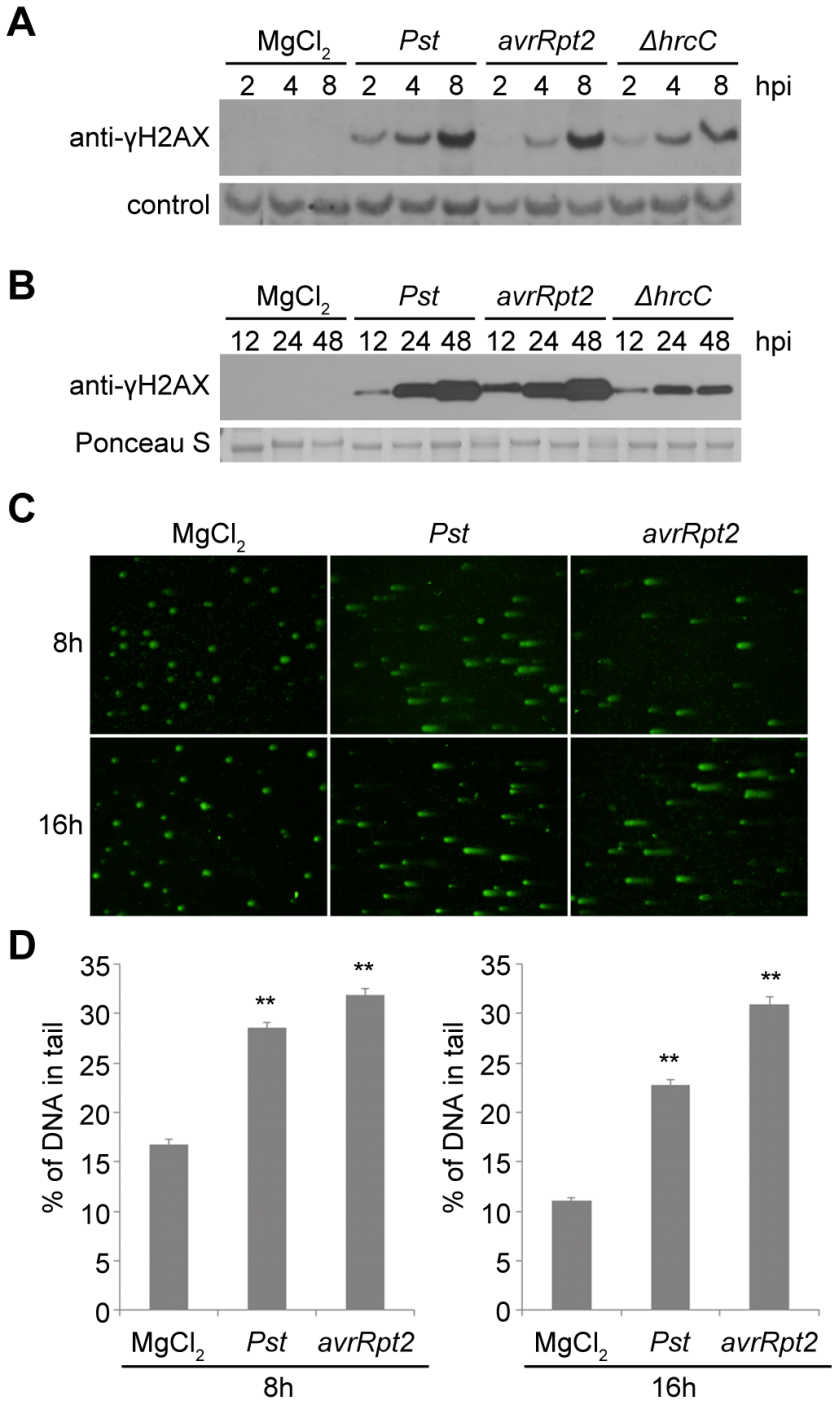
Host DNA damage by ***Pseudomonas syringae*** pv. ***tomato*** (***Pst***). (A–B) Accumulation of γ-H2AX during infection. Wild-type Arabidopsis Col-0 plants were vacuum-inoculated with (left to right) 10 mM MgCl_2_, *Pst* DC3000, *Pst* DC3000(*avrRpt2*) or *Pst* DC3000(Δ*hrcC*) at 1×10^7^ cfu/ml. The level of γ-H2AX was monitored at (A) 2, 4, 8 h, or (B) 12, 24, 48 h after inoculation, by immunoblot using anti-γ-H2AX antibody. Controls for equivalent loading included a non-specific band detected by the antibody (control) or Ponceau S staining of the same blot. Similar results were obtained in at least three separate experiments. (C) Representative *Pst*-induced DNA damage detected by comet assay. Wild-type Col-0 plants were inoculated with 10 mM MgCl_2_, or with *Pst* DC3000 or *Pst* DC3000(*avrRpt2*) at 1×10^7^ cfu/ml. Tissues were collected 8 or 16 h after inoculation and nuclei were subjected to comet assays. (D) Comet assay data presented as mean ± SE from at least 200 randomly selected nuclei for each treatment; data for 8 and 16 h are from separate experiments. **: significantly different from MgCl_2_-treated control (ANOVA *P*<0.01).
